# Evolution of Small Strain Soil Stiffness during Freeze-Thaw Cycle: Transition from Capillarity to Cementation Examined Using Magnetic and Piezo Crystal Sensors

**DOI:** 10.3390/s21092992

**Published:** 2021-04-24

**Authors:** Junghee Park, Jong-Sub Lee, Jongmuk Won, Jongchan Kim

**Affiliations:** 1 School of Civil, Environmental and Architectural Engineering, Korea University, 145, Anam-ro, Seongbuk-gu, Seoul 02841, Korea; chdl34@korea.ac.kr (J.P.); jongsub@korea.ac.kr (J.-S.L.); 2 Department of Civil and Environmental Engineering, University of Ulsan, Daehak-ro 93, Nam-gu, Ulsan 44610, Korea; jmwon@ulsan.ac.kr; 3 Department of Civil and Environmental Engineering, University of California at Berkeley, Berkeley, CA 94720-1710, USA

**Keywords:** capillary pressure, cementation, degree of water saturation, freeze-thaw cycle, water distribution patterns, elastic wave

## Abstract

Freeze-thaw cycles caused by seasonal temperature fluctuations significantly affect the geotechnical engineering properties. This study investigated the crucial role of water distribution patterns in the characterization of elastic wave properties for the fine F-110 sand during a freeze-thaw cycle. Sand specimens with four different water distribution patterns were prepared, namely homogeneously-mixed, evaporation-driven, vertically-, and horizontally-layered specimens. The *P*- and *S*-wave signatures of the specimens were monitored using piezo crystal sensors. Results indicated the criticality of water distribution patterns in the determination of small-strain soil properties even though the specimens had identical global water saturation. The nuclear magnetic resonance-based water volume depth profiles indicated that the evaporation-driven specimens had more heterogeneous pore-invasive ice-bonding layers at a high water saturation region; by contrast, the drying process facilitated uniform meniscuses around the particle contacts near the air percolation threshold. Elastic wave measurements for laboratory-prepared specimens might over/underestimate the small-strain soil stiffness of sediments in nature, wherein the drying processes prevailed to control the water saturation. This study highlighted a clear transition from capillary-controlled to cementation-controlled elastic wave properties during temperature oscillations.

## 1. Introduction

Sediments often undergo freeze-thaw cycles that typically originate from seasonal temperature fluctuations. These periodic changes in temperature result in the presence of an active layer at the near sediment subsurface that thaws during warm periods and freezes during cold periods [1]. Based on the particle-scale mechanisms, the active layer involves particle slippage, sliding, and rolling, followed by significant soil fabric changes that involve the rearrangement of soil particles, pores, and soil groups. Consequently, these freeze-thaw events in the active layer alter engineering soil properties, such as void ratio, permeability, compressibility, and strength. In particular, characterizations of sediments subjected to freeze-thaw cycles have enormous implications for cold region engineering and require multiphysics monitoring techniques [2].

Previous investigations employed low-perturbation elastic wave measurements to provide useful information required for enhanced soil characterizations and informative process monitoring during freeze-thaw cycles [3]. In addition, non-invasive methods aided in understanding the physical structure of frozen or thawed soils, including ice and unfrozen water contents [4,5,6,7]. At the particle scale, elastic wave velocities could successfully track the evolution of soil properties during phase transformation from water to ice, wherein significant changes in the small-strain stiffness were observed [8].

These significant changes mainly depended on the water saturation that determined the ice crystal volume, degree of ice bonding, and contact area between particles in frozen soils. Furthermore, the degree of water saturation affected the capillary pressure in sediments; therefore, the contribution of capillarity to effective stress-dependent wave velocities was significant, particularly in fine sands. A higher degree of water saturation tended to produce a slurry of soil particles with excess water flow during thawing. Clearly, the degree of water saturation appeared to be critical in the characterization and monitoring of soils using elastic wave measurement techniques during the freeze-thaw cycles. However, in nature, the heterogeneous water distribution in the pore space prevailed rather than the homogeneous distribution. Furthermore, the effect of water distribution patterns on elastic wave velocity during a freeze-thaw cycle remained unclear.

This study aimed to investigate the crucial role of water distribution patterns in the characterization of *P*- and *S*-wave properties for the fine F-110 sand during a freeze-thaw cycle. A modified oedometer cell was designed to house piezo crystals for *P*- and *S*-wave sensors and a thermocouple for temperature monitoring during the temperature oscillations, such as cooling, freezing, thawing, and heating. Fine sand specimens were prepared for different water distribution scenarios, such as homogeneous, evaporation-induced, vertically-layered, and horizontally-layered patterns at the target degrees of saturation, *S*, of 30% and 60%. Then, experimental data were analyzed using perfectly spherical particles and a nuclear magnetic resonance (NMR) device with respect to the water volume per specimen depth. The results indicated that laboratory measurements might over- or under-estimate the small-strain physical properties of sediments in the active layer, wherein evaporation processes played a dominant role in the determination of water saturation patterns in granular materials. This study highlighted the transition from capillary control to cementation control of the underlying mechanisms of elastic wave properties during a freeze-thaw cycle.

## 2. Materials and Methods

### 2.1. Tested Material—F-110 Sand

**Figure 1:**
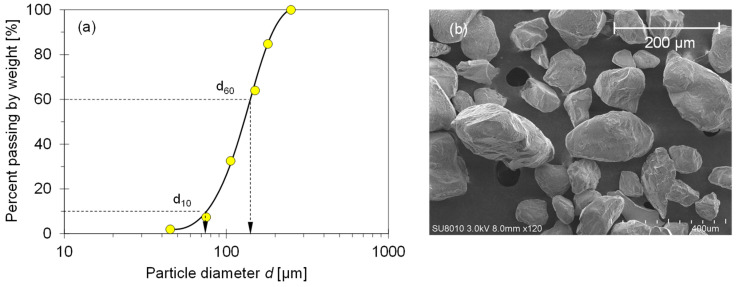
Characteristic of F-110 sand used in this study. (**a**) Grain size distribution curve. (**b**) Scanning electron microscopy (SEM) image.

This study employed well-qualified F-110 fine quartz sand for the experimental study. Figure 1 illustrates the grain size distribution curve and scanning electron microscope (SEM) image for the F-110 sand. This fine sand is the uniform-graded Ottawa sand that had a mean grain size (*d*_50_) of 120 μm, specific gravity (*G_s_*) of 2.65 (ASTM D854) [9], uniformity coefficient (*C_u_*) of 1.82, maximum void ratio (*e_max_*) of 0.85 (ASTM D4253) [10], and minimum void ratio (*e_min_*) of 0.54 (ASTM D4254) [11]. The reported roundness (*R*) of the F-110 sand was 0.70 [12].

### 2.2. Modified Oedometer Cell

**Figure 2:**
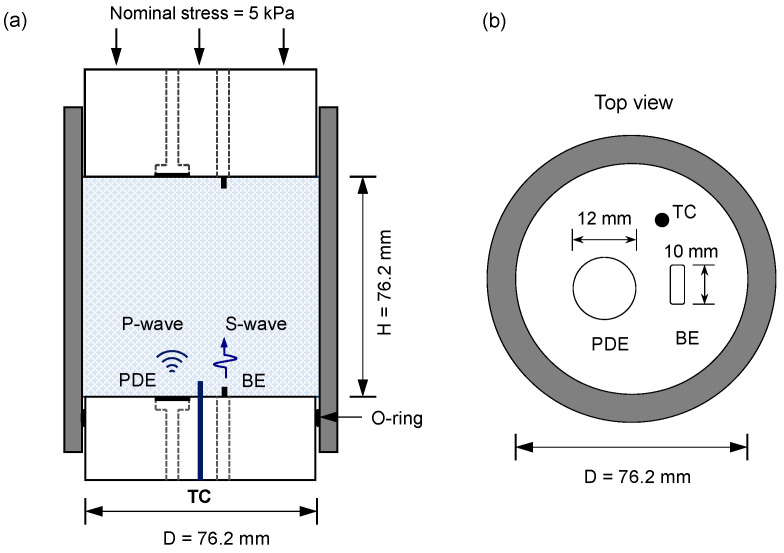
Schematic of the instrumented oedometer cell. (**a**) Side view of the assembled test apparatus. (**b**) Top view of the bottom cap. A pair of piezo disk elements (PDEs) and bender elements (BEs) was equipped for the *P*- and *S*-wave measurements, and a thermocouple was installed for temperature monitoring.

Figure 2 depicts a schematic of the instrumented oedometer cell. The transparent acrylic cell comprised three parts, namely top pedestal, main body, and bottom pedestal. The main body had an inner diameter of 76.2 mm and a wall thickness of 3 mm to ensure the zero-lateral strain condition (*ε_lateral_* ≤ 10^−5^) during the test. On the bottom and top pedestals, a pair of piezo disk elements (PDEs) and bender elements (BEs) was equipped as the *P*- and *S*-wave measurement sensors, respectively. A *T*-type thermocouple wire was installed on the bottom pedestal for temperature monitoring. Further details of the modified oedometer cell were described in [13].

### 2.3. Data Acquisition System

**Figure 3:**
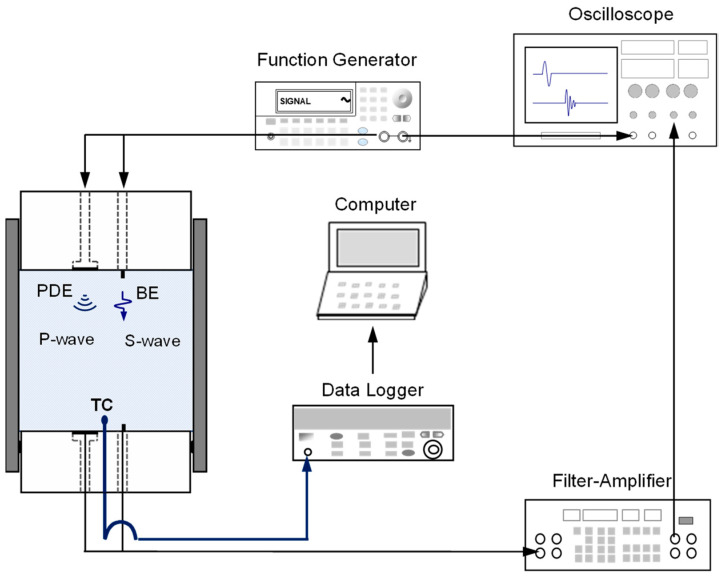
Configuration of measurement equipment for elastic wave and temperature monitoring.

Figure 3 illustrates the data acquisition system connected to the instrumented oedometer cell for *P*- and *S*-wave signature monitoring and temperature measurements. A function generator triggered the electric pulse signals to the top pedestal equipped with the PDE and BE. The parallel-type BEs used in this study minimized crosstalk [14]. The wave sensors converted the electric signals into mechanical vibrations, representing compressional propagation for the *P*-wave and shear propagation for the *S*-wave propagating through the specimen. The receiver sensors on the bottom pedestal detected the vibrations and converted them back into electric signals. Then, the electric signals were transmitted to the filter-amplifier for noise filtering. The received signals were filtered by low-pass filtering (L.P. = 2 MHz) and high-pass filtering (H.P. = 500 Hz). The processed signals were collected and displayed in an oscilloscope. The thermocouple TC for temperature monitoring was connected to a data logger. The specimen temperature was tracked every 10 s during the freezing and thawing processes.

### 2.4. Specimen Preparation

In this study, F-110 sand specimens were prepared with four different water distribution patterns, such as homogeneously-mixed, evaporation-driven, vertically-layered, and horizontally-layered specimens with target degrees of saturation of 30% and 60%. The four various water-distributed specimens were employed to represent the various possible water distribution scenarios in nature. First, F-110 sands were mixed homogeneously with deionized water (the amount of water was pre-calculated to attain the target water saturation *S* = 30% and 60%). The water-mixed sands were placed inside the oedometer cell and packed with a uniform tamping to obtain the homogeneous water-distributed specimens. These specimens were prepared with a relative density (*D_r_*, defined by ASTM D4254 [11]) of 40% and subjected to nominal vertical stress (*σ_v_*) of 5 kPa. Second, the evaporation-driven specimens were prepared by placing the fully-saturated sands in the oedometer cell. The saturated specimens with the target relative density were placed on a scale to track the water loss as the evaporation process progressed. The top pedestal was removed to expose the top surface of the specimens during the evaporation process. Once the amount of water remaining inside the specimens reached the target water saturation (i.e., *S* = 30% or 60%), the top pedestal was placed on top of the specimens with the nominal vertical stress. Third, the vertically-layered specimens were prepared with four ice-sand columns standing vertically, and the other spaces were filled with dry sand. These specimens comprised four high water content columns after the melting of the ice-sand columns, and the other spaces had lesser water content than the vertical water layers. Finally, the horizontally-layered specimens comprised three horizontal layers with dry–wet–dry sand zones in the cell (See detailed preparation methods for vertically- and horizontally-layered specimens [13]). The well-controlled water saturation resulted in a global degree of water saturation of 30% and 60% for all specimens.

### 2.5. Test Procedure

Following the preparation of specimens with various water saturation and distribution patterns, the oedometer cell was placed inside the freezer. The temperature of the freezer was set to −15 °C. The freezing process continued until no observable changes were detected in the *P*- and *S*-wave signatures, and then the frozen specimens were thawed by increasing the temperature to 18 °C. The vertical loading produced by the dead-weight on the top pedestal remained constant during the entire test procedure, resulting in equivalent vertical stress (*σ_v_*) of 5 kPa.

## 3. Results

Herein, the experimental results for the fine F-110 sand specimens were presented, including the temperature response and *P*- and *S*-wave signatures and velocities during a freeze-thaw cycle. The following section presented a comprehensive analysis of the dataset according to the water distribution patterns.

### 3.1. Temperature Response during Freeze-Thaw Cycle

#### 3.1.1. F-110 Sand with Different Water Saturations Prepared by Evaporation

**Figure 4:**
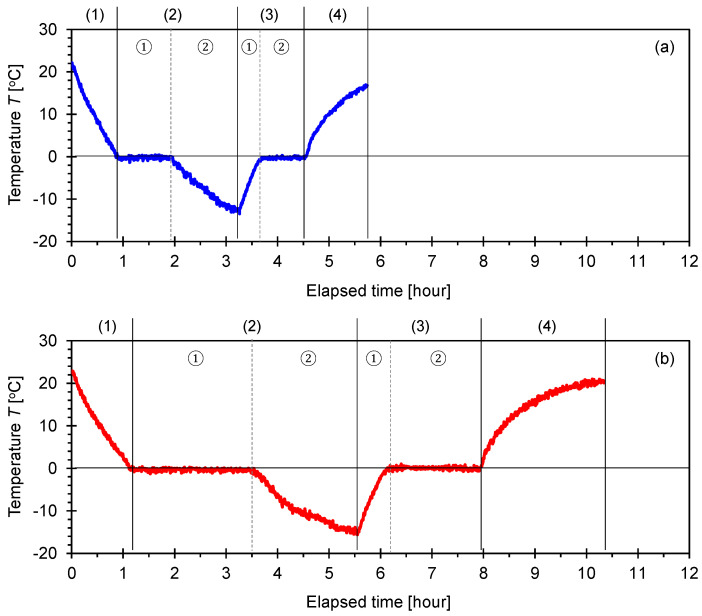
Changes in temperature during a freeze-thaw cycle for F-110 sand specimens with different degrees of water saturation prepared by the evaporation process. Time-dependent temperature involves (1) cooling; (2) phase change from water to ice; (3) thawing and phase change from ice to water; and (4) heating. (**a**) Degree of saturation *S* = 30% and (**b**) degree of saturation *S* = 60%.

Figure 4 depicts the changes in temperature with time during the freeze-thaw cycle monitored for the F-110 sand specimens with different degrees of saturation *S* = 30% and 60% prepared by an evaporation process.

For clarity, the time-dependent temperature response was divided into six zones to emphasize the consecutive cooling, freezing, thawing, and heating steps. Zone (1) corresponded to the cooling of the specimens from *T* = 20 to 0 °C. Zone (2)-① indicated that the temperature remained constant at *T* = 0 °C, where the bulk of the water in the pore space froze, and the transformation from the water to ice phase occurred. No supercooling process was observed. This exothermic reaction resulted in the duration of constant zero temperature (induction time), i.e., Zone (2)-① had a longer time period with a higher degree of saturation (similar trends were reported in [15]). Once the crystallization of ice occurred, the measured temperature decreased to *T* = −15 °C as shown in Zone (2)-② for further solidification. Warming began immediately after the temperature reached *T* = −15 °C (Zone (3)-①). The phase transformation from ice to water (i.e., thawing process) was an endothermic reaction and occurred at a constant temperature *T* = 0 °C (Zone (3)-②). In particular, the duration of the second zero temperature zone for the specimens with the degree of saturation of 60% was greater than that for the specimens with the degree of saturation of 30%. After the melting of the ice, heating to the room temperature *T* = 18 °C continued, resulting in the successful completion of the freeze-thaw cycle.

#### 3.1.2. F-110 Sand with Different Water Distribution Patterns

**Figure 5:**
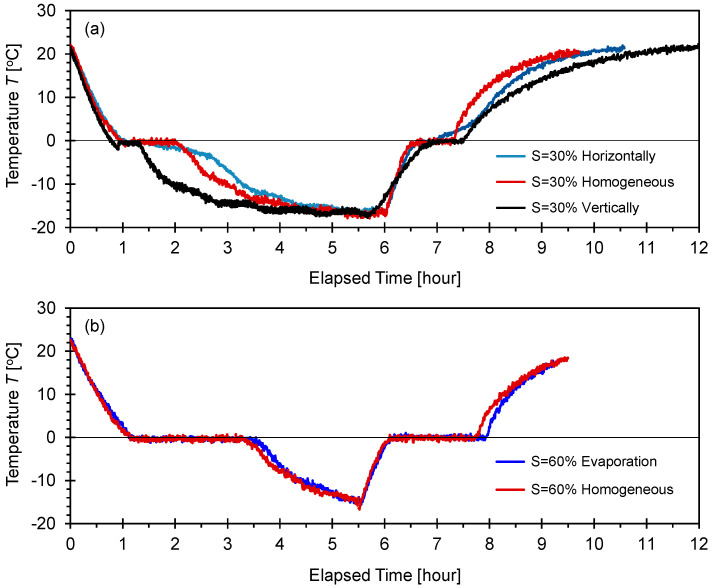
Changes in temperature during a freeze-thaw cycle for F-110 sand specimens with different water distribution patterns. (**a**) Degree of saturation *S* = 30% and (**b**) degree of saturation *S* = 60%. Time-dependent temperature involves (1) cooling; (2) phase change from water to ice; (3) thawing and phase change from ice to water; and (4) heating.

Figure 5 depicts the temperature response measured during the freeze-thaw cycle for F-110 sand specimens with various water saturation and distribution patterns. Each specimen exhibited a different water distribution pattern, including homogeneously-mixed, evaporation-induced, vertically-layered, and horizontally-layered water distributions for the target degrees of saturation *S* = 30% and 60%. For all specimens, the time-dependent temperature response consisted of six zones that resulted from cooling, freezing, thawing, and heating (similar trends can be observed in Figure 4). Figure 5a shows that the induction time varied with the water distribution patterns in the specimens at the degree of saturation *S* = 30%. However, the specimens with the degree of saturation *S* = 60% exhibited a significantly similar temperature trend with time, although the target water saturation was achieved through homogeneously-mixed and evaporation processes. The duration of the induction time during the freezing and thawing processes for the specimens with a degree of saturation of 60% was greater than that for the specimens with a degree of saturation of 30%.

### 3.2. Elastic Wave Signatures

**Figure 6:**
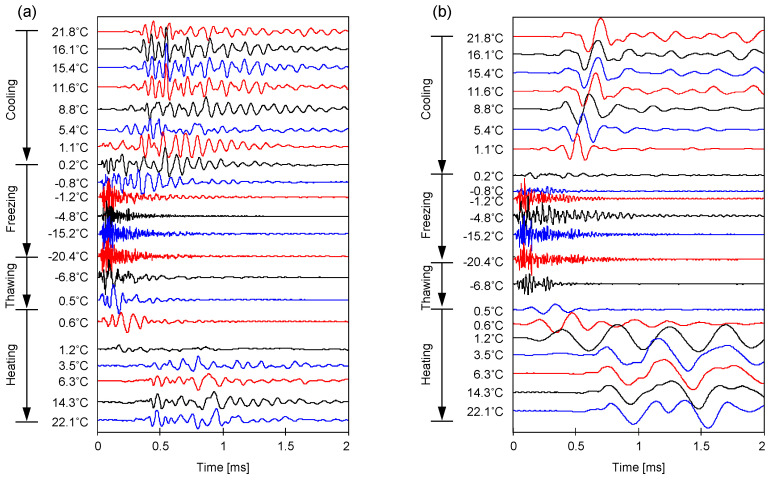
Elastic wave signatures measured during a freeze-thaw cycle for F-110 sand specimens at the degree of saturation *S* = 30% prepared by homogeneous mixing. (**a**) *P*-wave and (**b**) *S*-wave.

**Figure 7:**
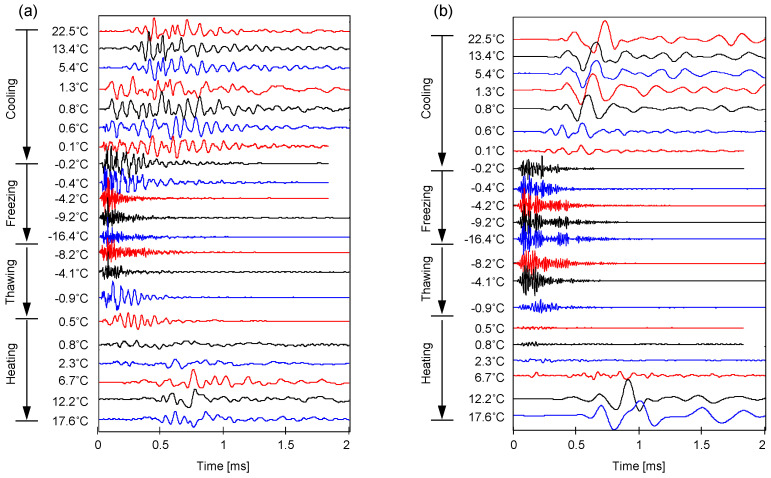
Elastic wave signatures measured during a freeze-thaw cycle for F-110 sand specimens at the degree of saturation *S* = 60% prepared by homogeneous mixing. (**a**) *P*-wave and (**b**) *S*-wave.

Figure 6 and Figure 7 depict the cascades of the captured *P*- and *S*-wave signatures of homogeneously-mixed specimens with degrees of saturation of *S* = 30% and 60%, respectively. The wave signatures were monitored during the cooling, freezing, thawing, and heating processes. The thermocouple installed on the bottom pedestal tracked the temperature of the bottom-end of the specimen. During the freezing process, the time to the first arrival for both the *P*- and *S*-wave signatures decreased. By contrast, the increase in temperature during the thawing process resulted in a significant increase in the first arrival time for both elastic waves, indicating a reduction in stiffness (similar observations were reported in [15,16,17]).

### 3.3. P- and S-Wave Velocities and Poisson’s Ratio during a Freeze-Thaw Cycle

#### 3.3.1. F-110 Sand with Different Water Distribution Patterns (S = 30%)

**Figure 8:**
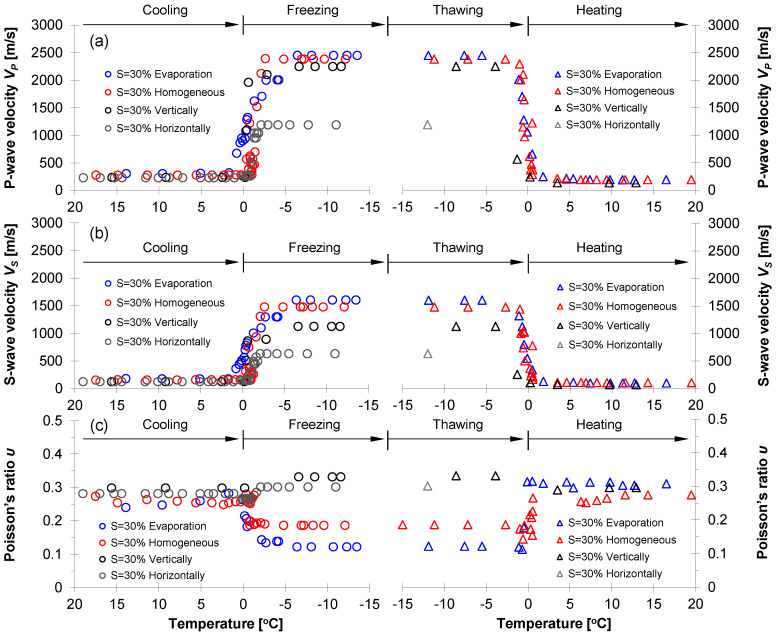
Evolution of small-strain stiffness properties during a freeze-thaw cycle for F-110 sand specimens at the degree of saturation *S* = 30% with different water distribution patterns. (**a**) *P*-wave velocity *V_P_*; (**b**) *S*-wave velocity *V_S_*; and (**c**) Poisson’s ratio *υ*.

Figure 8 shows the *P*- and *S*-wave velocities and Poisson’s ratio for four F-110 sand specimens prepared for various water distribution patterns at water saturation *S* = 30% (i.e., homogeneous, evaporation, vertically-, and horizontally-layered). Specifically, this study divided the entire test procedure into four categories: cooling, freezing, thawing, and heating. Before freezing (i.e., cooling process), although all specimens had identical global water saturation of *S* = 30%, the evaporation-driven specimens showed the highest *P*- and *S*-wave velocities and the horizontally-layered specimens had the lowest velocities (Figure 8a,b). The associated phenomena showed that the capillary force between particles built in the unsaturated condition contributed to increased effective stress; however, the capillarity varied with water distribution patterns [18,19,20,21,22]. The test specimens were prepared in the same manner except for the water distribution patterns. Therefore, the variation of *P*- and *S*-wave velocities before freezing suggested that the small-strain stiffness could be affected by not only water saturation but also water distribution patterns (Figure 8, [13,23]). A higher capillary force was expected in the evaporation-driven specimens in comparison to the force in the horizontally-layered case because the middle part in the horizontally-layered specimens had a relatively higher water saturation, i.e., *S* > 30%. The higher *P*- and *S*-wave velocities of the evaporation-driven specimens were clear evidence that the wave velocity was primarily affected by the effective stress.

Further temperature down to freezing, the *P*- and *S*-wave velocities for all specimens significantly increased due to the water-to-ice phase transformation. More complex stiffness enhancement might arise in pore-scale mechanisms where ice crystals emerged and interacted with particles. As the water transformed into ice, the ice might remain within pores or act as a bonding agent between particles. In fact, this ice pore habit depended on the degree of the water/ice saturation that determined the extent of cementation (=ice bonding) during freezing; the cementation effect became more pronounced with a higher water saturation because the pore water was more likely to be at most of the particle contacts followed by interconnected ice bonding skeletons. Since there were no water menisci available after the complete freezing process, the ice-particle bonding mechanism was the key factor determining the small-strain stiffness of the frozen specimens.

As the temperature increased from −15 ℃ to the melting point, the ice bonding effects reduced, and both *P*- and *S*-wave velocities decreased significantly. The comparison of *P*- and *S*-wave velocities before freezing and after thawing indicated that the freeze-thaw cycle lowered the *V_P_* and *V_S_* (i.e., see cooling and heating in Figure 8) because the temperature oscillation altered soil fabric and structure [24,25,26,27]. Further analyses in the context of ice pore habit and soil fabric were presented in later sections.

The measured *P*- and *S*-wave velocities enabled the estimation of the small-strain Poisson’s ratio *υ* using the following relation:

(1) $$\upsilon =\frac{0.5{\left(V_{P}/V_{S}\right)}^{2}-1}{{\left(V_{P}/V_{S}\right)}^{2}-1}$$

where *V_p_* is *P*-wave velocity and *V_s_* is *S*-wave velocity. Figure 8c shows the estimated Poisson’s ratio for four F-110 sand specimens with *S* = 30%. The Poisson’s ratio for unfrozen specimens during cooling ranged from 0.24 to 0.30, and the evaporation-driven specimens had the smallest Poisson’s ratio among all the specimens. With the decreasing temperature for the freezing process, the Poisson’s ratios for the evaporation-driven and homogeneous water mixing specimens decreased to 0.12 and 0.19, respectively (Poisson’s ratios for pure ice crystal and quartz were 0.33 and 0.17, respectively). The enhanced particle contacts and enlarged contact area due to the ice bonding in frozen specimens resulted in the decrease of the Poisson’s ratio for the two specimens. In particular, a more pronounced reduction in Poisson’s ratio was observed in the evaporation-driven specimen. As the ratio of *V_P_* and *V_S_* determined the Poisson’s ratio (Equation (1)), the increment rate of *S*-wave velocity before and after freezing was most pronounced in the evaporation-driven specimens. By contrast, the Poisson’s ratios for vertically- and horizontally-layered specimens increased slightly during freezing. Localized ice crystals concentrated in the wet vertical columns and horizontal disk layer result in increasing the wave velocities; however, the ratios of *V_P_* and *V_S_* for the two specimens were not susceptible to freezing. During heating, the Poisson’s ratios for all specimens changed to their initial values close to 0.30. Notably, the *P*- and *S*-wave velocities for the horizontally-layered specimens were not available due to uncertainty of the first arrival time after thawing.

#### 3.3.2. Homogeneously Mixed Method versus Evaporation (S = 60%)

**Figure 9:**
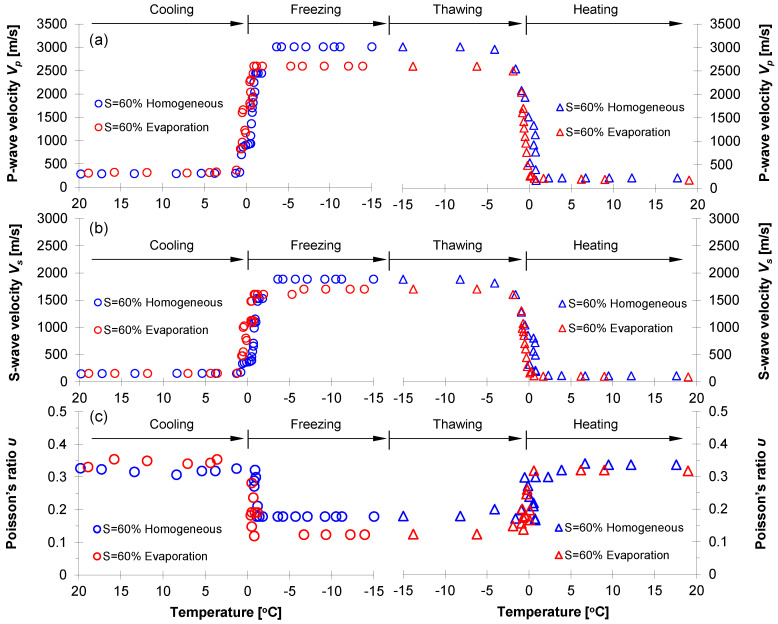
Evolution of small-strain properties during a freeze-thaw cycle for F-110 sand specimens at the degree of water saturation *S* = 60% prepared by homogeneously-mixed and evaporation process. (**a**) *P*-wave velocity *V_P_*; (**b**) *S*-wave velocity *V_S_*; and (**c**) Poisson’s ratio *υ*.

Figure 9 shows the *P*- and *S*-wave velocities and the calculated Poisson’s ratio for the evaporation-driven and homogeneously-mixed specimens where the target water saturation was *S* = 60%. During cooling at room temperature, the *P*- and *S*-wave velocities for both specimens were almost the same; however, the wave velocities increased sharply during the water-to-ice phase transformation and were much higher than the wave velocities in the case of water saturation *S* = 30%. All trends observed during a successful freeze-thaw cycle were similar to the trends observed in the case of water saturation *S* = 30% (see Figure 8). Detailed analyses were presented in the following sections.

## 4. Analyses and Discussion

This section reported a comprehensive analysis and discussion of the experimental results in the context of sediments’ temperature response, capillarity, water volume per unit depth, and the elastic wave properties during the transition from capillary to cementation.

### 4.1. Temperature Response

The pore-water temperature dropped below the freezing temperature before the water-to-ice phase transformation (i.e., supercooling). After ice nucleation, the release of latent heat reduced the temperature back to the freezing point, and it remained constant while the bulk pore-water froze (Figure 4 and Figure 5). In general, the freezing temperature of water in small pores was lower in comparison to the bulk water freezing temperature (i.e., freezing point depression) [28]. The Gibbs-Thomson equation explains the pore size-dependent freezing point depression ∆*T*_pore_ as a function of the pore diameter *d* such as ∆*T_pore_* = (4·*γ_wi_*·*T_bulk_*·cosθ_wi_)/(*ρ_w_*·∆*H*·d). Assuming that the interfacial tension between water and ice is *γ_wi_* = 26.7 mJ/m^2^, the freezing temperature of bulk water *T_bulk_* is 273 K, θ_wi_ is the contact angle when the water-ice interface is developed on the mineral surface, *ρ_w_* is the water density (=1000 kg/m^3^), Δ*H* is the specific latent heat of bulk water (=334 kJ/kg). The freezing point depression ∆*T*_pore_ anticipated in a fine-grained soil with pore diameter d = 0.1 μm is ∆*T*_pore_ ≈ −0.8 °C. Hence, phase transition in a fine-grained soil typically occurs at temperatures lower than 0 °C. By contrast, assuming the mean pore diameter *d* = 10 μm for the F-110 sand specimens, the anticipated freezing point depression is ∆*T*_pore_ ≈ −0.008 °C, which is negligible. This simple analysis confirmed the almost zero freezing point depression for the fine F-110 sand tested in this study (Figure 4 and Figure 5).

### 4.2. Anticipated Ice Pore Habit

From the sediment type point of view, a previous investigation provided X-ray CT images for frozen sandy and clayey specimens and revealed that the pore-invasive ice pore habit was dominant in coarse-grained soils, while grain-displacive ice growth, i.e., ice lenses, was relevant to fine-grained soils (refer to relevant investigations in [29,30]). Associated physics included a competing effect between effective stress and capillarity to determine the ice pore habit. The pore diameter for sand was relatively larger so that the effects of the capillary force ∆*u_wi_* = *γ_wi_*/*d* on the pore-habits were negligible. In fact, pore-filling ice growth was dominant in sands and led to a slight volume expansion together with minor changes in initial soil fabric during the freezing process. Generally, 9% volume changes were expected when the phase transformation between water and ice occurred. However, no observable volume changes during the freeze-thaw cycle were observed in this study when the tested specimens were in an unsaturated condition, i.e., *S* = 30% or 60%. The ice crystallization and growth could progress through near air-filled pore space rather than pushing particles away, resulting in volume expansion. Nevertheless, the minor alternations in localized or global soil fabric and particle contact features might occur during the freeze-thaw cycle. These changes that occurred during the freezing were not recovered after the thawing. This explained the lower *P*- and *S*-wave velocities after thawing in comparison to the wave velocities during the cooling process (Figure 8 and Figure 9).

### 4.3. Soil Water Characteristic Curve

**Figure 10:**
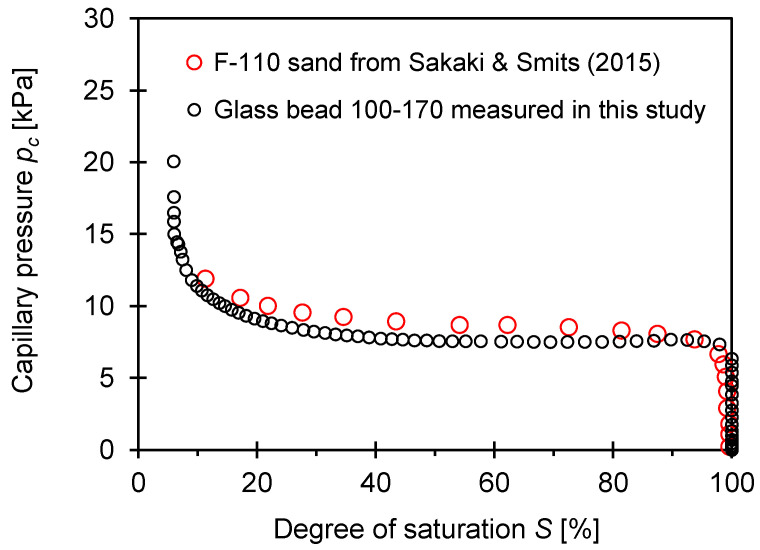
Soil water characteristic curve (SWCC) measured for glass bead 100–170. Note that the black open circle indicates the fine F-110 sand data extracted from [31].

This study employed a glass bead to investigate the effect of different sample preparation techniques on the experimental results. The perfectly-rounded glass bead 100–170 had a mean grain size *d*_50_ (=120 μm) similar to the tested F-110 sand; the effect of particle shape on the water distribution in pores was neglected in this study. For further comparison, Figure 10 shows the soil-water characteristic curve (SWCC) for the glass bead 100–170 measured in this study and the fine F-110 sand data obtained from the literature [31].

The capillary pressure against the degree of saturation for both materials was almost the same and decreased with a higher water saturation *S*. This water saturation-dependent capillary pressure played a critical role in determining the *S*-wave velocities at room temperature. In general, the *S*-wave velocities for unsaturated soils decreased with a higher water saturation because of the diminishing capillary pressure and the increasing bulk density (see details in [13,32]). Further analyses associated with capillary effects at room temperature were described in the following sections.

### 4.4. Water Saturation Profile with Depth

**Figure 11:**
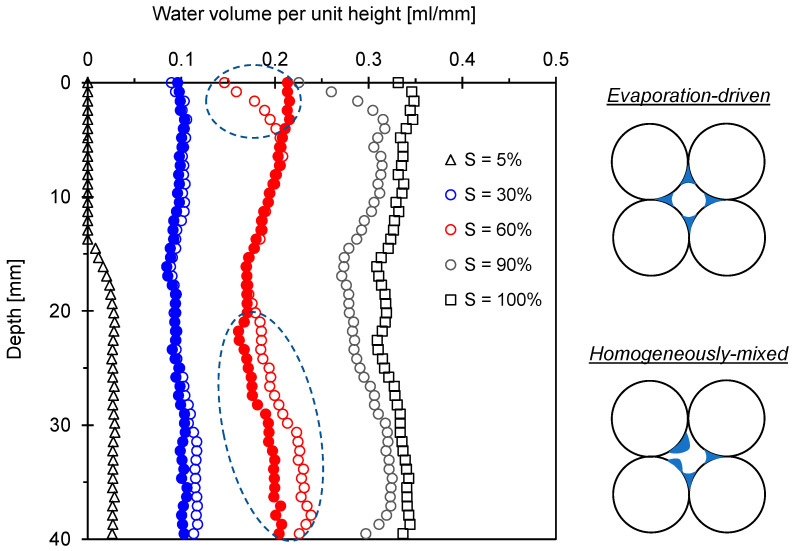
Depth-dependent water volume profile for the glass bead 100–170 measured using nuclear magnetic resonance (NMR). Note that open symbols indicate the specimens prepared by drying (evaporation) and closed symbols indicate the specimens mixed with water homogeneously.

Figure 11 shows the plot of water volume profile with depth for the glass bead 100–170 measured using an NMR device. Results showed that evaporation and homogeneous water mixing process at room temperature yield distinct water volume depth profiles in glass bead specimens. For the degree of saturation *S* = 60% prepared by an evaporation process, dry air blowing on the top of the specimen resulted in the rapid reduction in water content while the water content below the 20 mm depth was higher in comparison to the water content in the homogeneous water mixing specimen. This drying-induced water volume profile might result in more heterogeneous pore-invasive ice bonding layers during the freezing process; therefore, the *P*- and *S*-wave velocities for the frozen specimens at *S* = 60% prepared by evaporation were lower in comparison to the wave velocities in the homogeneously-mixed specimens (Figure 9a,b).

In contrast, the water volume depth profile for the two specimens at *S* = 30% was almost identical despite different specimen preparation methods. However, the drying process could help in the formation of the uniform meniscus between particles near the bulk water saturation corresponding to the air percolation threshold, while the homogeneous water mixing technique could cause the water to remain on the particle surface (refer to inset in Figure 11). Thereafter, water-to-ice phase transformation during freezing enhanced the bonding of particle contacts in the drying-induced specimen, and a higher cementation effect was expected. This pore-scale analysis reinforced the higher *P*- and *S*-wave velocities measured in drying-induced F-110 specimens in comparison to the wave velocities in the homogeneously-mixed specimens (Figure 8a,b). In fact, NMR-based depth-dependent water volume profiles indicated that elastic wave measurements for laboratory-prepared specimens might over- or under-estimate the small-strain soil stiffness of sediments in nature where drying process rather than homogeneous water mixing prevailed to determine the degree of saturation and water distribution patterns.

### 4.5. Transition from Capillary to Cementation

**Figure 12:**
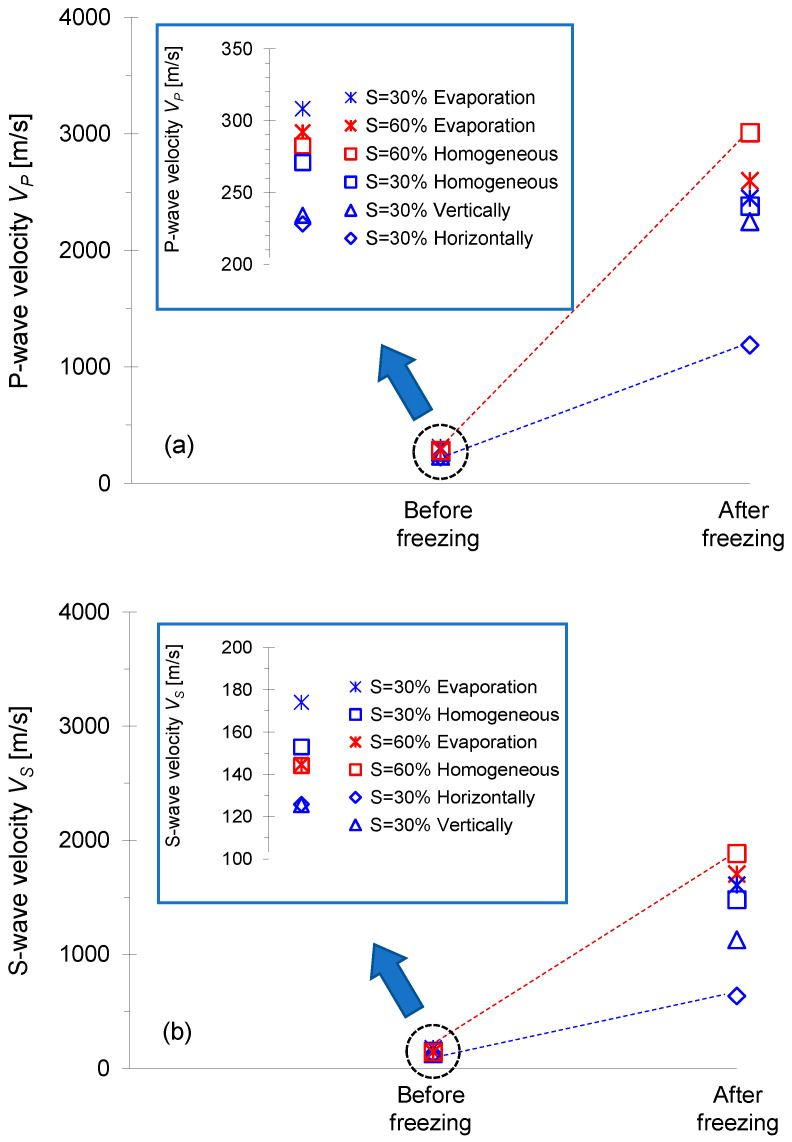
Transition from capillary-controlled to cementation-controlled elastic wave velocity during a freeze-thaw cycle. (**a**) *P*-wave velocity *V_P_*; (**b**) *S*-wave velocity *V_S_*.

Figure 12 shows the plots of the *P*- and *S*-wave velocities before and after freezing processes for all F-110 sand specimens tested in this study. Figure 12a shows that the *P*-wave velocity before freezing for the degree of saturation *S* = 30% prepared by evaporation was higher than the wave velocity of the two specimens with *S* = 60%.

For the specimens with *S* = 30%, the *P*-wave velocity before freezing varied with the water distribution patterns. The specimens prepared by evaporation showed the highest *P*-wave velocity, while the horizontally-layered specimens exhibited the lowest *P*-wave velocity. The *P*-wave readily propagated through the F-110 sand prepared by the evaporation process, where water meniscus uniformly formed between particles (refer to inset in Figure 11). In contrast, the *P*-wave passed through the dry–unsaturated–dry regions in the horizontally-layered specimens that hindered compressional wave propagation (Figure 12a). For the vertically-layered specimen, the relatively higher water-saturated columns facilitated the *P*-wave propagation without the effect of the dry sand zone present in the horizontally-layered specimen. There was a similar trend that reinforced the role of water distribution patterns on the shear wave velocity *V_S_* (Figure 12b). In particular, capillary pressure before freezing appeared to be critical in the determination of *V_S_* for the specimens prepared by the evaporation process and mixed homogeneously with *S* = 30%.

After freezing, a significant increase in *P*- and *S*-wave velocities for all specimens was observed. Clearly, a higher degree of saturation (i.e., *S* = 60%) resulted in more significant ice bonding and cementation between particles, followed by the greater *P*- and *S*-wave velocities in comparison to the velocities for *S* = 30%. However, the specimen preparation methods for *S* = 60%, i.e., evaporation and homogeneous water mixing, led to different water volumes with specimen depth, as shown in Figure 11. Therefore, homogeneous water mixing sand attained more uniform pore-invasive ice cementation that explains the highest *V_P_* after freezing. The *P*- and *S*-wave velocities after freezing exhibited the same trend with unfrozen specimens in view of different degrees of saturation (*S* = 30% and 60%) and various water distribution patterns. In particular, the *P*- and *S*-wave velocities for four frozen specimens with water saturation *S* = 30% indicated that the specimens with a higher wave velocity in the unfrozen condition showed a higher wave velocity after freezing. The beneficial role of the capillary forces on the unfrozen soil stiffness seemed to transfer to the ice cementation effect on the frozen specimen. In fact, there was a clear transition from capillary-controlled to cementation-controlled elastic wave velocity during a freeze-thaw cycle.

## 5. Conclusions

This study examined the critical role of water distribution patterns on the characteristics of elastic waves for the fine F-110 sand during the freeze-thaw cycle. A modified oedometer cell was designed to house piezo crystal sensors for *P*- and *S*-wave monitoring and a thermocouple for temperature tracking during the temperature oscillation. Fine sand specimens were prepared with different water distribution patterns such as homogeneous, evaporation-driven, vertically-, and horizontally-layered specimens at the target degrees of saturation *S* = 30% and 60%. All specimens placed into the freezer experienced cooling, freezing, thawing, and heating. The *P*- and *S*-wave signatures were monitored during the successful freeze-thaw cycle. Results were analyzed using the spherical glass bead and an NMR device in terms of water volume profile with depth. The main conclusions of this study were summarized as follows:

- Although sand specimens had identical initial global water saturation, i.e., either *S* = 30% or 60% at room temperature, the evaporation-driven specimen showed the highest *P*- and *S*-wave velocities, and the horizontally-layered specimen showed the lowest velocities because capillarity varied with water distribution patterns.
- Further temperature down to freezing, the *P*- and *S*-wave velocities for all specimens significantly increased due to the water-to-ice phase transformation. The cementation effect became more pronounced with higher water saturation.
- Pore-invasive ice pore habit existed in sandy soils, and no observable volume changes were observed in these unsaturated specimens. The slight decrease in *P*- and *S*-wave velocities after the thawing compared to the velocities in the initial cooling process suggested that minor alternations occurred in localized or global soil fabric and particle contact features where ice crystallization and growth was observed.
- The NMR-based water volume profiles with depth showed that the evaporation-driven specimen at *S* = 60% would have more heterogeneous pore-invasive ice bonding layers; therefore, elastic wave velocities for the frozen specimens prepared by drying were lower in comparison to the wave velocities for the homogeneously-mixed specimen.
- By contrast, the water volume depth profile at *S* = 30% was almost identical despite different specimen preparation methods. However, the drying process facilitated uniform meniscus around particle contacts, while the homogeneous water mixing technique led to the water partially remaining on the particle surface. This pore-scale analysis reinforced the highest *P*- and *S*-wave velocities measured in the drying-induced specimens in comparison to the wave velocities for the homogeneously-mixed specimen.
- In fact, elastic wave measurements for laboratory-prepared specimens might over- or under-estimate the small-strain soil stiffness of sediments in nature, where drying processes controlled the degree of saturation and formation of the meniscus.
- For F-110 sand specimens with the target water saturation *S* = 30%, the factor that determined small-strain soil stiffness before and after freezing was likely to transfer from the capillary force to ice cementation. In fact, there was a clear transition from capillary-controlled to cementation-controlled elastic wave velocity during a freeze-thaw cycle.

## Data Availability

The data presented in this study are available on request from the corresponding author.
